# Locally Relevant High‐Resolution Hydrodynamic Modeling of River Floods at the Regional Scale

**DOI:** 10.1029/2021WR030820

**Published:** 2022-07-01

**Authors:** Andreas Buttinger‐Kreuzhuber, Jürgen Waser, Daniel Cornel, Zsolt Horváth, Artem Konev, Michael H. Wimmer, Jürgen Komma, Günter Blöschl

**Affiliations:** ^1^ VRVis Zentrum für Virtual Reality und Visualisierung Forschungs‐GmbH Vienna Austria; ^2^ Institute of Hydraulic Engineering and Water Resources Management Vienna University of Technology Vienna Austria; ^3^ Department of Geodesy and Geoinformation Vienna University of Technology Vienna Austria

**Keywords:** inundation modeling, river floods, flood hazard mapping, large‐scale hydraulic modeling

## Abstract

This paper deals with the simulation of inundated areas for a region of 84,000 km^2^ from estimated flood discharges at a resolution of 2 m. We develop a modeling framework that enables efficient parallel processing of the project region by splitting it into simulation tiles. For each simulation tile, the framework automatically calculates all input data and boundary conditions required for the hydraulic simulation on‐the‐fly. A novel method is proposed that ensures regionally consistent flood peak probabilities. Instead of simulating individual events, the framework simulates effective hydrographs consistent with the flood quantiles by adjusting streamflow at river nodes. The model accounts for local effects from buildings, culverts, levees, and retention basins. The two‐dimensional full shallow water equations are solved by a second‐order accurate scheme for all river reaches in Austria with catchment sizes over 10 km^2^, totaling 33,380 km. Using graphics processing units (GPUs), a single NVIDIA Titan RTX simulates a period of 3 days for a tile with 50 million wet cells in less than 3 days. We find good agreement between simulated and measured stage–discharge relationships at gauges. The simulated flood hazard maps also compare well with local high‐quality flood maps, achieving critical success index scores of 0.6–0.79.

## Key Points

- Second‐order accurate scheme discretizing the full shallow water equations at a resolution of 2 m for a stream network of 33,880 km length
- Streamflow adjustment for regionally consistent flood peak probabilities and automatic estimation of boundary conditions for arbitrary domain tiling
- Model accuracy comparable to local models (critical success index scores of 0.6–0.79)

## 1 Introduction

Climate change has modified river floods in Europe (Blöschl et al., 2019) and other parts of the world (Alfieri et al., 2017; B.Merz et al., 2021). In order to assist flood management agencies, regional planners, and insurance companies in adapting to the changing hazard, large‐scale inundation maps associated with a given return period are needed. Recently, extensive research has been carried out on continental and global inundation mapping (Alfieri et al., 2014; Bates et al., 2021; Dottori et al., 2016; Sampson et al., 2015), but bridging the gap between continental scale and local models is still challenging because of limitations regarding simulation accuracy and cost.

First, the accuracy of the hydraulic simulation is controlled, among other factors, by the numerical scheme and the resolution of the computational domain. Resolutions of a few meters are required to resolve terrain features such as levees and small channels. However, they require an efficient processing of a large amount of possibly heterogeneous data for large areas. Traditionally, two‐dimensional (2D) hydrodynamic simulations were considered unviable for areas larger than 1,000 km^2^ and resolutions at the meter scale (Teng et al., 2017) due to the high computational costs, even though the value of a high resolution is clear (Ernst et al., 2010; Fewtrell et al., 2011; Horváth et al., 2020; Shustikova et al., 2019; Xia et al., 2019). Various approaches have therefore been proposed for speeding up simulations. One example are linked 1D/2D models (Falter et al., 2016; Hoch et al., 2019; Morales‐Hernández et al., 2013; Rajib et al., 2020), but they have disadvantages related to complex data preprocessing, implementation of the linking model and the need for a case‐by‐case decision if the linked model is indeed significantly faster than a 2D model. Another possibility consists of simplifying the physical processes, for example, neglecting advection or inertia terms (Neal et al., 2012). While in general this seems to be a computationally very efficient alternative, efficiencies may drop for urban regions (Costabile et al., 2020) and for receding flows including wet‐dry boundaries (Cozzolino et al., 2019). Cozzolino et al. (2019) conclude that numerical issues in these problematic regions may originate from simplifying the shallow water equations (SWEs) while discretizations of the full SWEs are not subject to these limitations. Second‐order finite‐volume (FV) schemes (Audusse & Bristeau, 2005; Hou et al., 2015; Murillo et al., 2008) or discontinuous Galerkin (DG) schemes (Kesserwani et al., 2008; Shaw et al., 2021; Vater et al., 2017) offer additional accuracy, however at the cost of higher runtimes. For large‐scale river flooding, the usage of a second‐order scheme instead of its first‐order counterpart may be preferrable in view of the workload–accuracy tradeoff (Horváth et al., 2020). Perhaps more important is the reduction of simulation runtimes by code parallelization. In particular the use of graphics processing units (GPUs) may lead to a drastic reduction of runtimes (Brodtkorb et al., 2012; Echeverribar et al., 2019; Horváth et al., 2016; Vacondio et al., 2016) when compared to central processing units (CPUs). Simulations of flood events on GPUs may be faster than realtime for domains of hundreds of km^2^ and resolutions of a few meters (Morales‐Hernández et al., 2021; Xia et al., 2019).

Second, an efficient approach to simulating large regions with numerous streams is needed. Typically, a single processing unit in a computer cluster, or a so‐called computational node, is not able to accommodate the entire domain. Thus the entire domain needs to be split into subdomains with their sizes bound by the computational capability, for example, memory, of the individual computational node. Common approaches involve a decomposition of the domain into individual river reaches allowing only small changes in flood discharges within the subdomains (Alfieri et al., 2014; Bates et al., 2021; Sampson et al., 2015). At the boundary of the subdomains, streamflow hydrographs are usually prescribed as inflow boundary conditions (BCs). In flood hazard mapping, the peaks of the streamflow hydrographs typically correspond to the flood quantiles of the return period of interest. For large simulation tiles, however, changes of the river flood quantiles along the stream network within the subdomain can no longer be ignored because of lateral inflows of tributaries and diffuse sources. Moreover, at confluences the downstream flood quantile is not simply the sum of the upstream quantiles as the upstream flood quantiles are typically not fully correlated in space.

Third, the accuracy of the inundated areas also depends on the accuracy of hydrologic and topographic input data, dense upstream streamflow boundary conditions (Rajib et al., 2020), and detailed models to capture local flow dynamics. Local relevance is thus not only achieved by high spatial resolution, but also by the inclusion of small‐scale local features. For example, underpasses usually appear closed in digital terrain models (DTMs), but may actually be open during a flood event. The inclusion of levees and dams is required to ensure that protected areas remain dry in the inundation model (Bates et al., 2021; Wing et al., 2017). Also, the way buildings and structures are treated has an effect on the small‐scale dynamics of the water flow (Dottori et al., 2013). Culverts and power plants are often included in local models, but usually neglected in large‐scale models. Inclusion of these structures is needed for locally relevant, regional flood simulation, especially in densely inhabited areas. The accuracy of the models can be assessed by comparing the flood areas with carefully designed local models (Wing et al., 2017), with remotely sensed flood inundation extents (Rajib et al., 2020), with observed high water marks (Wing et al., 2021), and with insurance claims (Zischg et al., 2018).

Fourth, multiple steps are required for setting up the input data and the BCs for hydraulic simulations. In a traditional modeling setup, these steps are usually carried out manually to allow for the handling of special cases which is almost always occur in real‐world applications. An automatic execution of the workflow requires a consistent approach to process the steps, for example, a dataflow consisting of linked submodels that share data via input and output connections. Ideally, the whole modular dataflow is controlled by one interactive automation framework (Sampson et al., 2015) that allows for localized data corrections and automatically triggers only local resimulations.

**Figure 1:**
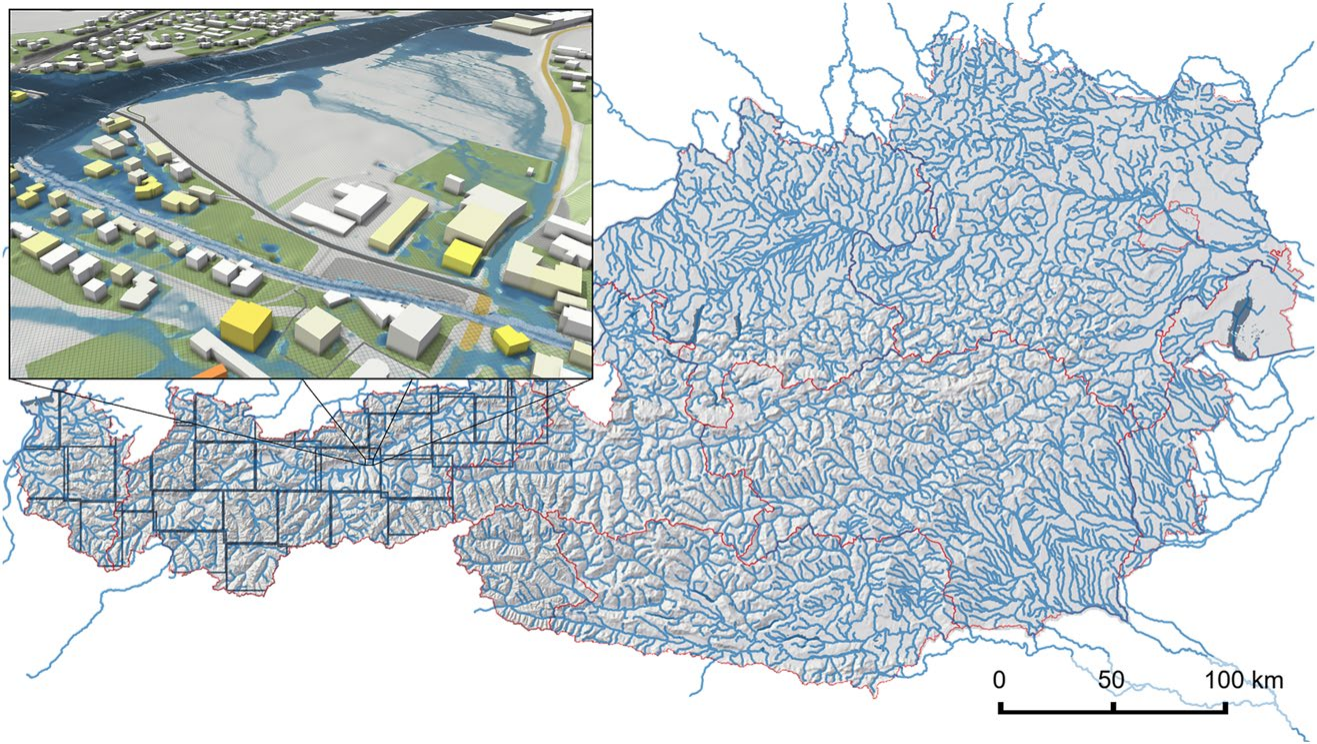
Simulated rivers in Austria with decomposed simulation domains shown in the west. The inset image shows the simulated maximum water depths on the 2 m grid. The high resolution together with regionally consistent flood quantiles result in detailed, locally relevant inundation maps.

In this paper, we present a framework for simulating inundated areas representing the river flood hazard in large domains at a high resolution. The study area is Austria (84,000 km^2^) and the resolution chosen is 2 m (Figure 1). This paper goes beyond the existing literature by simulating a river network of 33,880 km with a second‐order scheme that discretizes the full SWEs. We demonstrate the effectiveness of accelerated computational models to enhance the accuracy and local relevance of the simulations. We propose a novel method to ensure consistent flood quantiles across the entire river network of large simulation domains. Starting from raw input data for an entire country, we describe an automated simulation setup that derives BCs on‐the‐fly for the derivation of inundated areas. The proposed model introduces streamflow hydrographs for all catchments greater than 10 km^2^ and accounts for levees, buildings, and culverts to provide locally relevant flood hazard maps. We also perform a combined validation against rating curves of stream gauges and against detailed local flood maps.

## 2 Methods

### 2.1 Integrated Setup as a Visdom Dataflow

**Figure 2:**
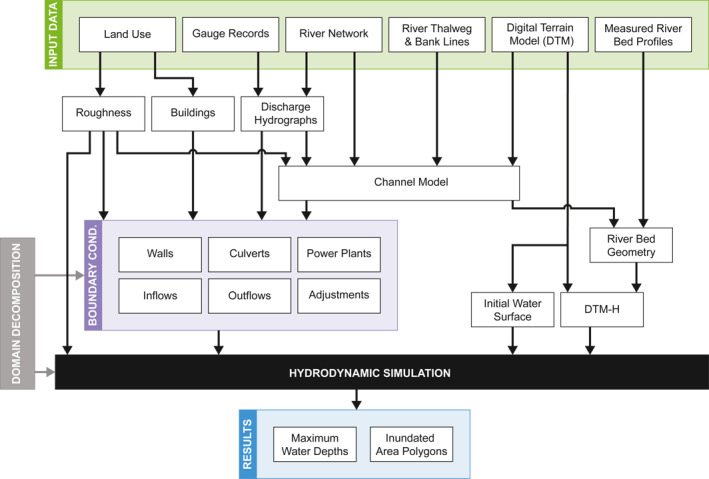
Simplified dataflow of the flood inundation model in the proposed automation framework.

While local simulation studies are often set up in an ad hoc way to maximize flexibility and cater for local requirements, larger scale studies require a more formal approach given the larger data volumes, in particular if different types of data are to be combined in an efficient way (Federal Emergency Management Agency, 2019; Sampson et al., 2015). Since the focus of this study was on maximizing the synergies of using local information with simulations at large scales, we are proposing a data flow that allows a combination of automatic and manual processing steps and at the same time ensures consistency of different data types. In order to obtain inundation maps from raw input data, a cascade of submodels, the so‐called modular dataflow, needs to be executed. A simplified version of the dataflow used in our approach is shown in Figure 2. The raw input data comprise land use, stream gauge records, the river network, river thalweg and river bank lines, the DTM, and measured river bed profiles, as shown at the top of Figure 2.

For a given simulation domain, the dataflow then assembles all the necessary data for the hydraulic simulation including the roughness, the boundary conditions (BCs), the hydrologically enforced digital terrain model (DTM‐H), and an initial state of water levels. The roughness is estimated from land use in the floodplains and from calibrated roughness at stream gauges (Section 2.2.5). The BCs account for buildings and hydraulic structures, such as culverts and power plants. Moreover, at the boundary of the simulation domain and at river origins, the BCs prescribe flood hydrographs (Sections 2.2.3 and 2.3). This sub‐dataflow is visualized on the left of Figure 2.

The DTM‐H represents a DTM that has been prepared for hydraulic modeling, for example, bridges have been cut‐out and river beds have been burned in to allow free flow (Section 2.2.1). For most of the larger rivers in Austria bed measurements exist. Thus, we choose a dual approach. Where measurements exist, the river bathymetries are used to modify the DTM. Where no measurements exist, we estimate a trapezoidal profile based on discharge, the river network topology, the river thalweg and the river bank lines, and the DTM (Section 2.2.4). This sub‐dataflow is visualized on the right of Figure 2.

The entire study region is split manually into rectangular tiles (Section 2.3.1). There are no restrictions on the domain decomposition from the input data. Given any domain, instationary BCs are specified automatically. The only step involving manual manipulations are corrections of data errors, for example, of river bed measurements or the coordinates of river thalweg and bank lines. The manual corrections and the domain decomposition are stored as “actions” in separate layers which are later applied automatically to the relevant data. For each of the tiles, BCs are automatically generated and the computation of the required input data for the hydrodynamic simulation is triggered. Initial water depths for the hydraulic simulation are obtained by subtracting the DTM‐H from the original DTM. The simulation then computes water depth fields and velocity vector fields. Finally, at the bottom of the cascade, the flood hazard maps are identified from the simulated maximum water depths (Section 2.4).

Ideally, the execution of the dataflow should not involve any manual intervention. Here we use the interactive automation framework Visdom (Schindler et al., 2013; Waser et al., 2011), which determines the execution order by itself and executes the cascade of submodels in a fully automated way. Moreover, Visdom features an interactive visual representation of the dataflow consisting of several dozens of modular units and hundreds of connections. In reality the dataflow is more complex than the illustrative dataflow shown in Figure 2. Visdom allows interactive changes to the dataflow in an accessible graphical user interface throughout the entire project period. Moreover, the graphical representation greatly facilitates the comprehension of the dependencies between submodels.

### 2.2 Input Data and Pre‐Processing

#### 2.2.1 Terrain Model

The digital terrain model (DTM) covers all of Austria with a spatial resolution of 1 m and is based on light detection and ranging (LIDAR) data. The vertical precision of airborne laser scanning (ALS) is at the order of a few centimeters (Kraus, 2011; Pfeifer & Briese, 2007). However, the accuracy of the DTM‐H, which is derived from the DTM, also depends on other factors not fully represented by ALS data, for example, water bodies and vegetated river banks. The acquired point clouds completely lack information about submerged topography, such as river beds, which has to be added. Bridges and non‐monotonic terrain levels along rivers are eliminated from the DTM and the gaps are filled by adaptive river course interpolation between the unobstructed parts to obtain the DTM‐H (Wimmer et al., 2021).

#### 2.2.2 River Network

**Figure 3:**
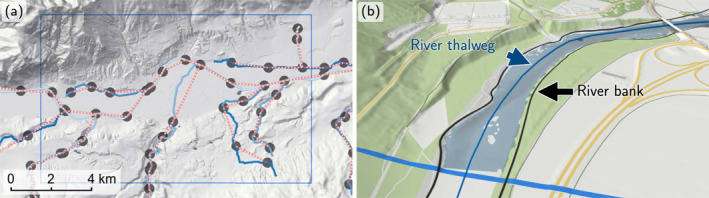
(a) River network around Innsbruck, Austria. The network consists of nodes (gray circles) linked by the river. The nodes hold the hydrologic data, such as flood discharges. (b) Detail of river thalweg lines and river bank lines.

The digital river network consists of nodes and their directed connections. Each node is allowed to only have one downstream node. Nodes are placed at catchment outlets, at confluences (Figure 3a), and where stream discharges change significantly, for example, downstream of power plants. The 19,479 nodes hold the hydrologic data, such as flood discharges. The river thalweg lines are automatically realigned with thalweg lines derived from the DTM. In cases of doubt, manual checks and revisions are performed (Wimmer et al., 2021). Additionally, a left and a right river bank line representing the extent of the water body in case of bank‐full flow are generated (Figure 3b). In total, 33,880 river km are delineated representing all Austrian streams with a catchment size greater than 10 km^2^.

#### 2.2.3 Streamflow Boundary Conditions

While in simulations of physical events, streamflow boundary conditions are given by observed or synthetic flood discharge hydrographs (Grimaldi et al., 2013), hazard simulations as in this study use effective flood discharge hydrographs. The hydrographs may take any shape as far as the simulation model is concerned, but there is value in making them representative of the multiple flood events that occur in a reach and estimate them from observed streamflow data. Here we assume hydrographs of the form (Dottori et al., 2009)

(1) $$Q\left(t\right)=Q_{B}+\left(Q_{T}-Q_{B}\right){\left(\frac{t}{t_{P}}\exp\left(1-\frac{t}{t_{P}}\right)\right)}^{\gamma },$$

where *Q*_*B*_ is the mean annual discharge, *Q*_*T*_ is the discharge of the flood peak for the associated return period, for example, *T* = 100 years, and *γ* is set to 3 based on hydrograph analyses in the study area. The time to peak parameter *t*_*P*_, *Q*_*B*_ and *Q*_*T*_ are estimated by a statistical regionalization method based on Top‐Kriging (Skøien et al., 2006) following R. Merz et al. (2008).

#### 2.2.4 River Bed Geometry

**Figure 4:**
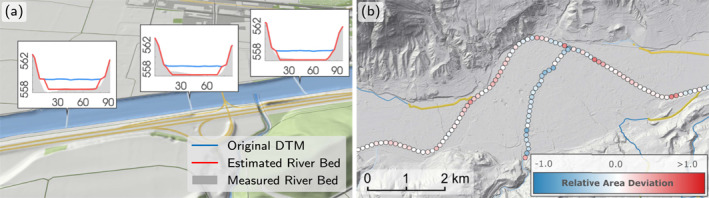
(a) Example validation of the estimated trapezoids (red) burned into the original digital terrain models (DTM) (blue) against the measured bottom topography (gray) at the Inn river. Numbers are length across the river (m) and level (m). (b) Comparison of the burn‐in approach against measured cross‐sectional data along the Inn river and a tributary in terms of their relative wet area deviation. Red indicates an overestimation of the computed wet areas, blue an underestimation. The yellow lines indicate culverts, where the river bed was not burned in.

In Austria, 18.6% of all river km are covered by measured profile data, mostly at large rivers. Following Bures et al. (2019) and Fleischmann et al. (2019), we adopt a combined approach and construct the river bed from measured river bed profiles and estimated trapezoidal cross‐sections. The cross‐sectional river width is based on the distance between the left and right river bank lines. The depth is determined by Manning's equation under the assumption of a trapezoidal cross‐section with a bank slope of 60°. The Manning's roughness value is set to the same value as used in the SWEs (see Section 2.2.5). The slope of the energy grade line is determined by sampling the original DTM including water bodies along the river thalweg line. The discharge consistent with the time of the DTM survey is assumed to be proportional to the mean annual discharge. A proportionality factor of 0.7 was backcalculated from measured profile data. A factor smaller than 1 is in line with the practice of conducting DTM surveys in autumn when stream flow is low in Austria (R. Merz et al., 1999). Figure 4a shows a comparison of estimated trapezoidal cross‐sections and measured river bed geometries for a reach of the Inn river and Figure 4b shows the associated relative wet area deviations defined as the ratios of estimated wet areas over measured wet areas. For the major river flowing from west to east (the Inn river) the bed is approximated very well. For the southern tributary, the estimated cross‐section is slightly too deep.

#### 2.2.5 Roughness

Accurate estimates of roughness are essential, as simulated velocities, and thus water levels and inundation extent, are usually quite sensitive to channel roughness (Hall et al., 2005; Savage et al., 2016; Werner et al., 2005). In general, roughness can be calibrated from observed water levels or inferred from landscape characteristics (such as landuse), using published relationships (Arcement & Schneider, 1989; Chow, 1959). Here the roughness coefficient is compiled from different sources, in an attempt to maximize its information content.

**Figure 5:**
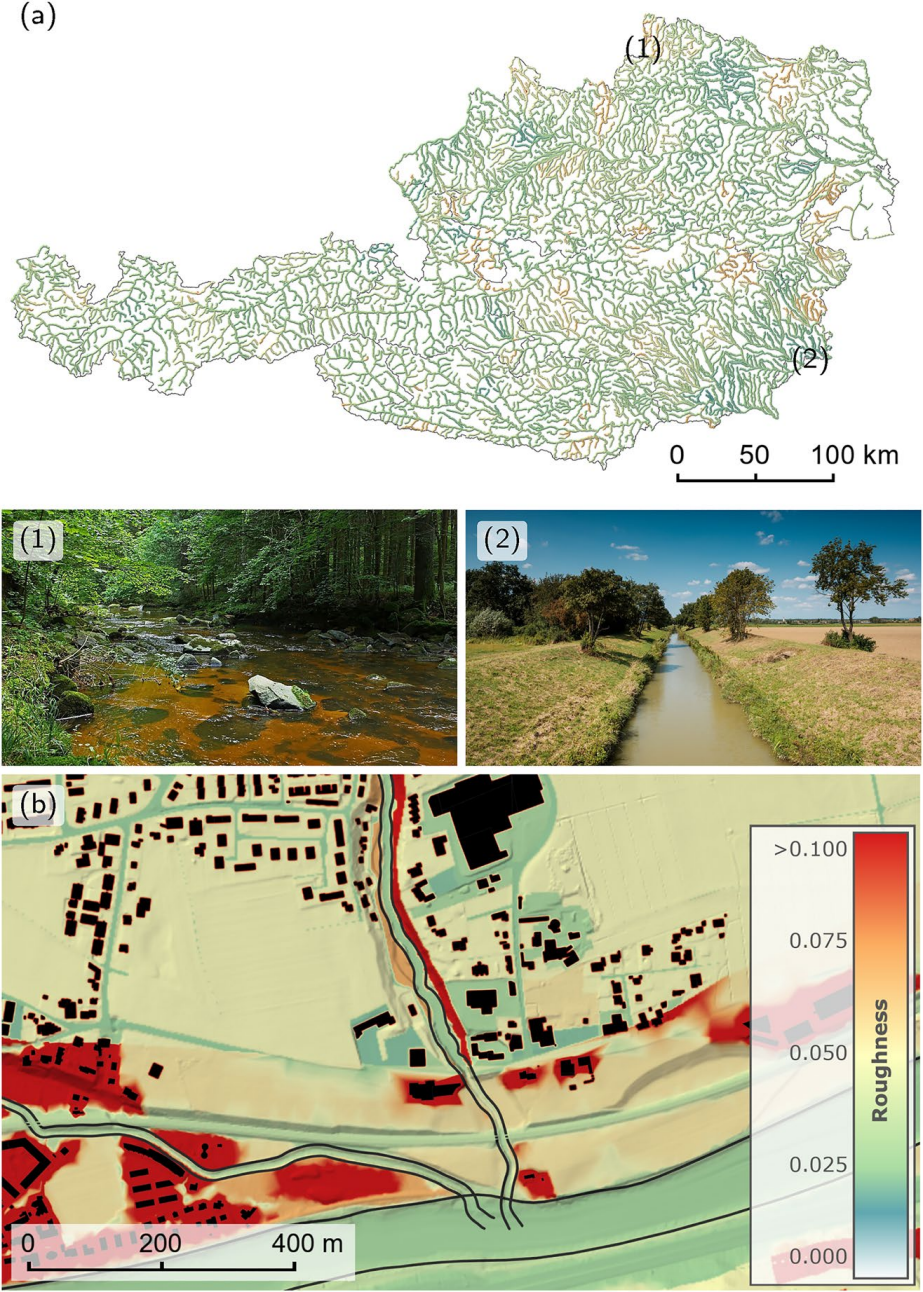
(a) Roughness coefficients in the river channel interpolated from coefficients calibrated at the stream gauges to the river network with Top‐Kriging. The photos give an impression of the river characteristics of locations with (1) a high roughness of around 0.06 s/m^1/3^ for a natural stream in rocky hills and (2) a relatively low roughness of 0.02 s/m^1/3^ for a regulated stream in the lowlands. (b) The spatially distributed roughness coefficients in the floodplains are based on land use. The roughness legend is valid for both (a) and (b). Image Copyright: (1) https://commons.wikimedia.org/wiki/File:Die_Lainsitz_im_Gabrielental_bei_Weitra_01_NDM_GD-103.jpg, CC BY‐SA 3.0 AT. (2) Hydro Burgenland, Heiligenbrunn/Strem. Permission granted.

In a first step, floodplain and river channel are treated separately. On the floodplains, a mapping from land use type to Manning roughness coefficients is adopted from Chow (1959). Inside the river channel, we calibrate the roughness with water level and discharge data from 420 stream gauges. We choose to calibrate the roughness at each gauge individually with Manning's equation, in line with the estimation of the trapezoidal cross‐sections and the rating curves at the outflows. The approach of individual calibration is computationally much more efficient than a joint calibration on all the gauges, because of its lower dimensionality. The calibrated roughness values are interpolated on the river nodes with Top‐Kriging. To reduce the influence of only locally valid high roughness coefficients at some gauges, we average the estimated roughness values with a default roughness value of 0.03 s/m^1/3^, which is the median over the entire stream network. The resulting Manning roughness coefficients along all rivers are displayed in Figure 5a.

In a second step, roughness coefficients from previous local studies are incorporated if available for a reach, which is the case for around 10% of the river network (3,350 river km). As the roughness coefficients from local studies are often calibrated based on local data, there is an extra benefit beyond the information from the first step. However, they sometimes contain artefacts and inconsistencies in overlapping river reaches. In order to ensure an overall homogeneous treatment and at the same time take advantage of local information, the roughness coefficients from local studies are averaged on a cell‐by‐cell basis with the ones based on land use (in the floodplains) and the ones interpolated from the stream gauges (in the river channels). The averaging method adopted is a pragmatic choice to give the information from previous local studies equal weight to that of the present one. A detail of the final spatially distributed roughness field is shown in Figure 5b.

### 2.3 Automatic Boundary Conditions

#### 2.3.1 Tiling

As the simulation operates on a uniform Cartesian grid, rectangular tiles are an efficient and natural choice for decomposing the study region. The region is split into 182 rectangular simulation tiles on the basis of the following criteria: The tiles are specified in such a way that power plants are far enough away from an inflow, so that backwater effects are fully captured. Cities and large confluences are sought to be in the middle of a tile to avoid boundary effects. The tiles are required to overlap with each other to capture all flooded areas originating from levee overtoppings several kilometres up‐ or downstream in the case of large rivers. To balance these goals, the tiles are specified manually. Although the tiling process could be automated, we argue that a manual approach, in which the various controls can be evaluated in a flexible way, may be more efficient, given that there are only 180 tiles to specify.

**Figure 6:**
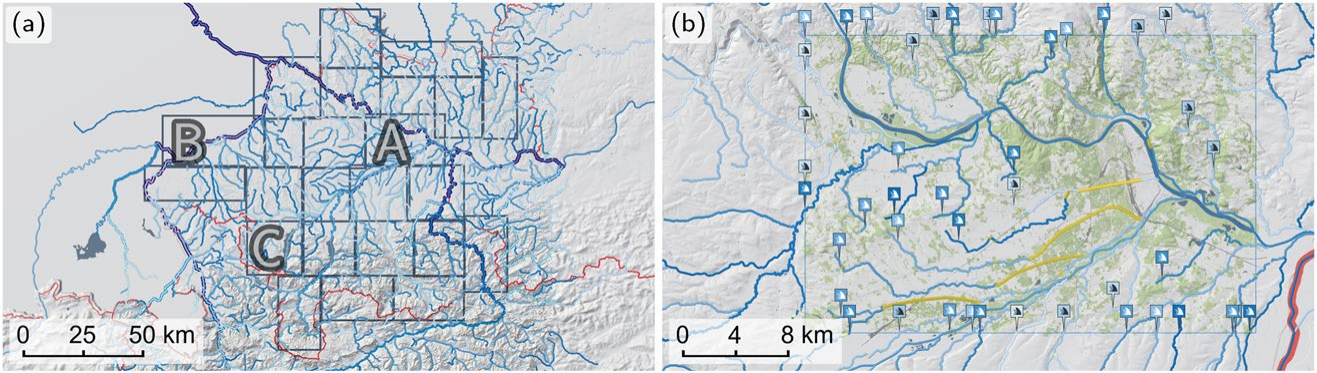
(a) Domain decomposition of Upper Austria into 24 tiles which are simulated in parallel. The size of a tile is limited by the memory of a single GPU. The tiles labeled A, B, C show slow simulation performance (Figure 11). (b) Close‐up of tile A with all BCs. Inflows and outflows are displayed as wave icons. Culverts are indicated as dark yellow lines.

The tile size is limited by the available memory on the GPU to a total number of around 150 million wet cells. Thus, given a resolution of 2 m, we limit the tiles to an area of 1,000 km^2^, assuming a maximum of 60% wet cells. An example tiling is shown in Figure 6 along with the locations of the automatically derived boundary conditions, including inflows and outflows shown as wave icons. The tiles are simulated completely independently of each other enabling fast parallel processing.

#### 2.3.2 Automatic Inflows

**Figure 7:**
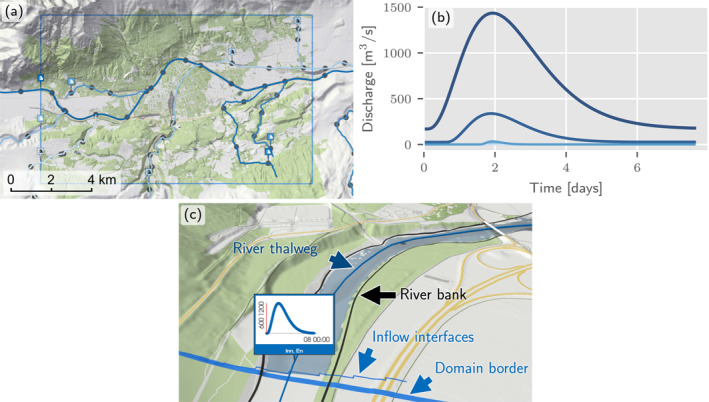
Automatically derived inflows for an arbitrarily chosen domain. (a) Inflows are placed at the start of rivers and when streams enter the domain. (b) At each of the nine inflows shown in (a), a flood hydrograph is calculated from the interpolated hydrologic parameters of the upstream node. (c) At an inflow, the discharge is then distributed on the rasterized inflow interfaces of the cross‐sectional line normal to the river thalweg.

Whenever a river enters a simulation tile or starts inside a simulation tile, upstream BCs are derived (Figure 7a). The flood hydrograph at the inflow positions is calculated by Equation 1 from interpolated hydrologic parameters (Figure 7b). In order to account for the travel times of the flood waves, the hydrographs are shifted in time by a recursive algorithm (Section 2.3.4). This is because synthetic, effective hydrographs are simulated. Such a shift would not be needed if observed events were simulated.

The inflow geometry is identified by constructing a cross‐sectional line normal to the river line and intersecting it with the river bank lines. This line is extended by a fixed factor on both sides, and based on trial and error a factor of two was chosen (Figure 7c). At the rasterized cell interfaces of the extended cross‐sectional line, we prescribe a fraction of the discharge given by the hydrograph. The fraction at each interface is assumed to be proportional to the water depth ensuring a natural distribution of the discharge, as only neighboring wet cells receive a water influx.

#### 2.3.3 Streamflow Adjustments for Consistent Quantiles

Flood hazard mapping requires the estimation of inundation areas that conform to the same return period everywhere. Based on the definition of a T‐year flood as the discharge exceeded with a probability of 1/T in any one year at a specific point on the stream network (Fuller, 1914), the corresponding inundation areas also conform to the same return period, if a unique, monotonic relationship exists between water level and discharge. The inundation areas are thus associated with the same return period in a region (Alfieri et al., 2014; Bates et al., 2021; Sampson et al., 2015). Previous studies have sometimes approximated these inundation patterns by simulation of real events, for example, with the hydrograph peaks of the desired return period along the main stream, and smaller return periods at the tributaries as may occur during real events (Sampson et al., 2015), but this approximation is not amenable to the simulation of large regions where the same return period of the peak flows should be maintained everywhere. This is because an infinite number of hydrological situations can produce a T‐year flood discharge at a specific point. A conceptually more consistent alternative are long‐term simulations of many events over hundreds of years (e.g., Falter et al., 2016), from which the inundation areas associated with a return period T can be determined by extreme value analysis, but these long‐term simulations are computationally very expensive. Here, we propose a new, computationally more efficient method that allows maintaining the same return period of flood peaks on the entire stream network for effective hydrographs with a peak flow corresponding to a T‐year return period. This effective hydrograph does not represent one event but the summary effect of all possible events. Since the flows associated with the same return period along the stream network are not mass conserving along gaining reaches and at river junctions, water is added or removed in a precisely specified way.

**Figure 8:**
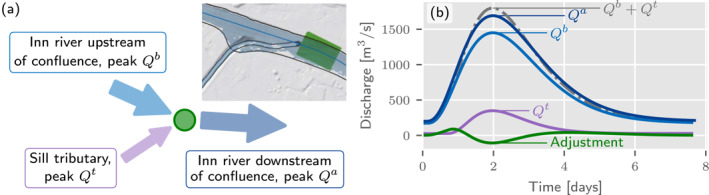
Hydrographs at the Inn–Sill confluence for a 100‐year flood. (a) A schematic illustration of the confluence with an inset image of the real‐world situation. (b) The hydrographs at the confluence with the hydrograph of the tributary shown in violet. The adjustment is made at specific cells (indicated in green in (a)) via a source term.

Small tributaries not explicitly resolved in the river network and diffuse lateral inflows contribute to an increase in flood discharges along a river. In such a case, water is added along the river to ensure return periods are maintained. Confluences are considered in a similar fashion. Such a situation is illustrated in Figure 8a for the Inn–Sill confluence. In Figure 8b the corresponding hydrographs of the tributary before the confluence, as well as the hydrographs of the main river before and after the confluences, are plotted. The sum of the peak discharge of the tributary and that of the main river upstream of the confluence is generally greater than the peak discharge downstream of the confluence. This is because of the probability of a joint occurrence of floods at both streams is usually significantly smaller than 1 (Bender et al., 2016; Guse et al., 2020). For a confluence node, the adjustment is given by the difference between the sum of the upstream hydrographs and the downstream hydrograph associated with that node. The resulting difference hydrograph at the confluence is negative around the peak time (Figure 8b). Therefore, at a confluence typically water needs to be removed from the hydraulic simulation to ensure the return period is maintained. This adjustment is performed at rasterized adjustment polygons related to the river geometry (indicated in green in Figure 8a). In these cells, a specific source term is introduced in the SWEs, which applies the adjustment to the wet cells in the polygon.

The proposed approach ensures that inundated areas are not underestimated before confluences, as in other approaches, where the flood quantile at the outlet is redistributed to the inflows in the same tile (Sampson et al., 2015). The adjustment serves as an hydrological‐hydraulic interface and is applicable to any hydrograph shape. For continuous hydrological models (Falter et al., 2016; Rajib et al., 2020) or for individual events (Costabile & Macchione, 2015) the difference hydrographs are always non‐negative, even after confluences, and the adjustments are to be interpreted as diffuse lateral inflows.

#### 2.3.4 Temporal Relationship Between Streamflow Hydrographs

At each river node, a hydrograph is specified by the statistical regionalization described in Section 2.2.3 with the peak flow representing the discharge of a given return period and the volume estimated as the median of observed events. In addition, some assumption is needed regarding the timing of the hydrographs. We propose the following procedure to provide temporal relationships between streamflow hydrographs for an efficient mapping of the inundated areas within a simulation tile.

Within a river reach without tributaries, the wave travel time between two neighboring nodes is estimated by a uniform wave approximation and the velocity obtained from Manning's law using the median water depth, the average roughness and slope along the reach as well as the river width, assuming the wave celerity is not vastly different from the flow velocity.

**Figure 9:**
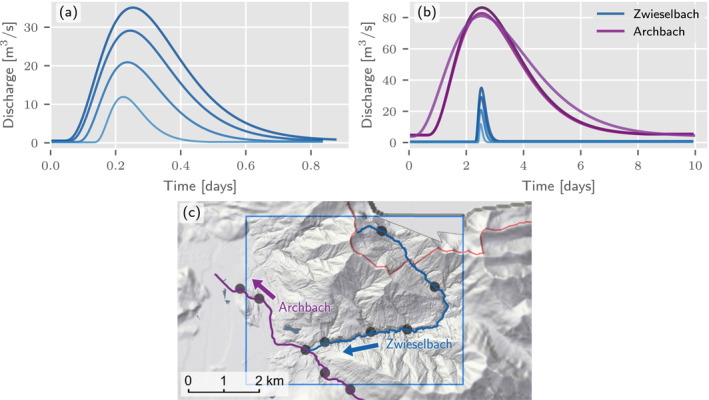
(a) Streamflow hydrographs at the river network nodes of a single river reach, the Zwieselbach in Tyrol. The peaks are slightly shifted by time lags computed from a uniform wave approximation. (b) Streamflow hydrographs at the nodes of the main river, the Archbach, and a tributary, the Zwieselbach. At the confluence the peaks are exactly aligned. The river with the shorter hydrograph duration, in this case the Zwieselbach, is shifted toward the river with the longer hydrograph duration. (c) Associated nodes and domain.

At confluences, the timing difference of the tributaries cannot be inferred from hydraulic relationships. This is because the simulations do not represent a real event but the summary effect of many events that may have very different characteristics, mainly due to differences in the spatial rainfall distribution. We therefore assume that flood waves at confluences peak simultaneously. This assumption ensures that the differences in the discharge time series are smaller than for non‐synchronous flood peaks and it enhances numerical efficiency, as any non‐zero timing difference between the tributary and the main river increases the duration of the simulation. We illustrate the effects of the travel time approximation and the synchronous peaking assumption at confluences in Figures 9a and 9b, respectively, on a small domain shown in Figure 9c. Alternative formulations could be based on the bivariate statistics of the coincidence of floods at river confluences (Bender et al., 2016; Guse et al., 2020).

If a simulation tile is divisible into multiple smaller independent river networks, the peak time at the outflows is set to occur at the same time. This rule increases simulation performance as it ensures fast simulations until the common flood peak, as the simulation is faster before the flood peak than after the flood peak due to the smaller percentage of wet areas.

Based on the above assumptions, a recursive algorithm estimates all the time shifts needed for the adjustment and inflow hydrographs. The required simulation time of a tile is controlled by the node with the maximum hydrograph duration. The hydrograph duration is determined by the time to peak parameter and the travel time needed for the wave at that node to leave the domain. The required simulation durations range from 1.7 days in steep terrain to 31 days in tiles with lakes and large rivers.

#### 2.3.5 Rating Curve Outflows

**Figure 10:**
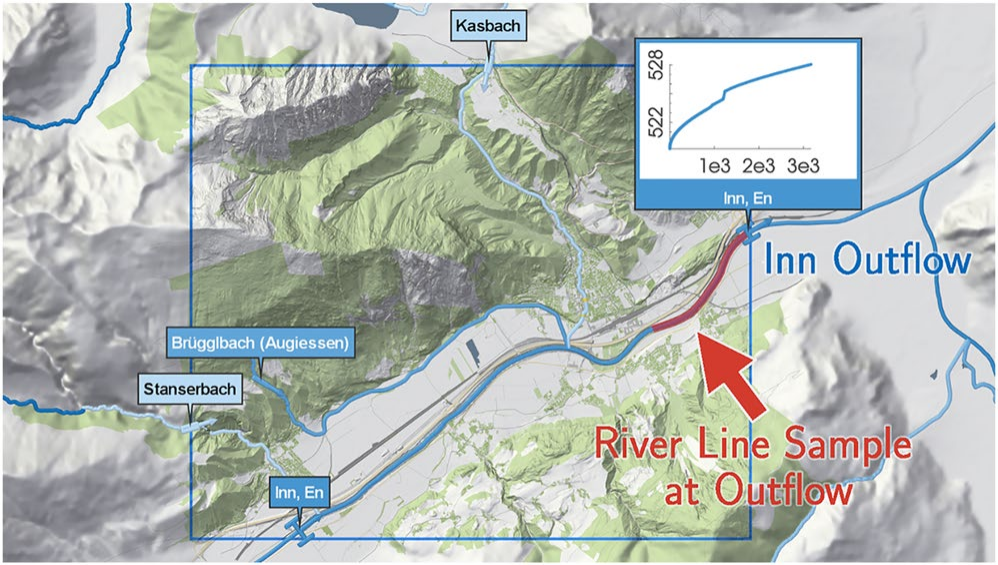
Outflow rating curve automatically derived from the water level slope along the river thalweg line, the roughness along the river and along the outflow cross section. Simulated outflow discharges are mapped to a water level at every time step.

Whenever a river leaves a tile, outflow BCs are automatically derived. The geometric setup is similar to the inflows, however we extend the cross‐sectional line only by 20% at each side in contrast to a 100% extension for inflows. We specify an absorbing BC at the domain boundary allowing water to freely leave the domain. Thus, if water bypasses the specified outflow interfaces, it can leave the domain at the boundary. To ensure numerical stability, we prescribe a dynamic water level BC at the outflows. Specifically, we employ a rating curve to dynamically map the simulated discharge that leaves the domain through this outflow BC to the corresponding water level at every time step (Figure 10). Assuming uniform flow, we use Manning's equation to derive the rating curve. We emphasize that *dynamic* is intended to mean that the water level time series is not a priori set from recorded gauge data, but computed at every time step via the rating curve. This dynamic outflow condition ensures a correct specification of the water level, accounting for changes of the flood wave inside the simulation domain, such as peak discharge reduction due to dike overtoppings or floodplain spills, and avoids a possibly incorrect propagation of a priori converted peak water levels at the downstream boundaries into the simulation domain.

#### 2.3.6 Buildings, Culverts and Power Plants

Given the resolution of 2 m, all buildings in the domain are rasterized as wall cells where no water can enter. In urban regions, culverts and sewers may affect the inundation area. While the representation of urban sewer systems is beyond the scope of this paper, we do use a simple culvert model supporting culverts without bifurcations that are fed by streams and aligned with river thalweg lines. The cross‐sectional geometry is automatically derived from the river thalweg and river bank lines at the ends of a culvert. The discharge is computed taking into account the pressure heads and the cross‐sectional geometry at the in‐ and outlet. In total, there are 1,475 culverts.

Additionally, run‐of‐river and high‐head power plants are considered. For large rivers, the weirs of run‐of‐river power plants that are opened during floods are represented in the DTM‐H. The retention effects of high‐head power plants are simulated by specifying water level‐discharge relationships of the outlets. Downstream of the reservoirs, at the outlets, hydrographs are set as upstream BCs analogously to the inflows in Section 2.3.2.

### 2.4 Hydrodynamic Simulation

The hydrodynamic simulation engine solves the full two‐dimensional shallow water equations (SWEs)

(2) $$\partial_{t}U+\partial_{x}F\left(U\right)+\partial_{y}G\left(U\right)=S_{b}\left(U,b\right)+S_{f}\left(U\right)+S_{c},$$

where $U={\left[h,hu,hv\right]}^{T}$ is the vector of conserved variables and **F** and **G** are the flux functions

(3) $$F=\left[\begin{array}{c} hu\\ hu^{2}+\frac{1}{2}gh^{2}\\ huv \end{array}\right],\;G=\left[\begin{array}{c} hv\\ huv\\ hv^{2}+\frac{1}{2}gh^{2} \end{array}\right].$$

The bed slope term **S**_*b*_ models the fluid's acceleration due to gravitational forces,

(4) $$S_{b}=\left[\begin{array}{c} 0\\ -gh\partial_{x}b\\ -gh\partial_{y}b \end{array}\right].$$

Flow resistance is modeled by the friction term **S**_*f*_,

(5) $$S_{f}=\left[\begin{array}{c} 0\\ -gn^{2}h^{-1/3}u\sqrt{u^{2}+v^{2}}\\ -gn^{2}h^{-1/3}v\sqrt{u^{2}+v^{2}} \end{array}\right].$$

In these definitions, *h* represents the water height, *hu* is the discharge along the *x*‐axis, *hv* is the discharge along the *y*‐axis representing the conserved variables. Furthermore, *u* and *v* are the average flow velocities in *x* and *y*‐directions respectively, *g* is the gravitational constant, and *b* is the bathymetry (assumed to be time‐independent), and *n* is the Manning roughness coefficient. The source and sink term **S**_*c*_ is only active in adjustment cells with prescribed positive or negative discharges (Section 2.3.3).

For the spatial discretization of the SWEs, the finite volume method (FVM) is used on a uniform Cartesian grid of 2 m. We use a second‐order accurate, shock‐capturing finite‐volume scheme (Buttinger‐Kreuzhuber et al., 2019). The benefit of a shock‐capturing second‐order scheme relative to a first‐order scheme has been highlighted by several studies for small regions, for example, for steep catchments (Kvočka et al., 2015) and for large‐scale studies in combination with fine resolutions (Dazzi et al., 2021). In some subregions of the simulation domain of the present study, Horváth et al. (2020) has shown that the water level errors of a second‐order finite‐volume (FV2) scheme are less than half of those of its first‐order counterpart (FV1). We believe that the results of the subareas extend to the entire simulation domain because of a similar topography, similar event magnitudes and similar simulation tile sizes. Although a FV2 scheme requires around 5 times longer runtimes than a FV1 scheme, in the present study the FV2 scheme was therefore preferred.

The scheme preserves still‐water and lake‐at‐rest steady states and properly handles flow states across bed discontinuities. Second‐order accuracy in space is achieved through a minmod limiter. The minmod parameter is set to one, in order to ensure robust and fast simulations (Horváth et al., 2020). At wet‐dry boundaries only the velocities are set to zero below a cut‐off water depth threshold of 0.0001 m, thus ensuring mass conservation up to floating‐point precision. The friction source term **S**_*f*_ is evaluated in a semi‐implicit manner by splitting it into a coefficient‐wise product of an implicitly evaluated state and an explicitly evaluated friction term (Brodtkorb et al., 2012). For the second‐order time integration we use Heun's method (Buttinger‐Kreuzhuber et al., 2019).

The FVM enables straightforward parallelization on regular grids. The scheme is implemented on GPUs using the CUDA platform (Wilt, 2013) for substantially faster runtimes relative to CPUs (Brodtkorb et al., 2012; Morales‐Hernández et al., 2021; Xia et al., 2019). On a GPU, parallel tasks are organized into blocks. A block size of 12 by 12 cells is used as it is the optimal block configuration for second‐order schemes allowing for a high utilization of the GPU (Horváth et al., 2016). The implementation is heavily optimized and data dependencies are handled with on‐chip memory for fast processing of the computational stencil. For fuller details the reader is referred to Horváth et al. (2016). Each simulation tile is assigned to a specific GPU and split into thousands of blocks. When a block is dry and not at risk of flooding, it is excluded from the hydraulic simulation. Therefore, only wet blocks have an impact on the simulation runtime. Well‐constructed datastructures consisting of mostly single‐precision floats guarantee that the GPU achieves maximum performance. We use 10 NVIDIA Titan RTX GPUs in parallel, each equipped with 24 GB of video memory. For the second‐order accurate scheme, each GPU is able to process a maximum of approximately 150 million wet cells. The exact number depends on the distribution of the wet cells, input data size of the BCs and other minor factors.

The tiles specified in Section 2.3.1 are processed in parallel and independently of each other. Each GPU processes one tile and does not need to exchange data with another simulation. For a cluster of 10 GPUs, 10 tiles are processed in parallel. Whenever a GPU is ready for work, after finishing its previous job, the next tile in the queue is assigned to it automatically by the dataflow system (Section 2.1). Once all tiles are processed they are aggregated. Since the tiles are simulated independently, the inundated areas do not necessarily agree in overlapping regions. In these regions, the water depths of the overlapping tiles are averaged with weights depending on the distance from the tile boundary ensuring smooth transitions at tile borders. This results in a unique maximum water depth for every location of the study domain. Finally, polygons of the inundated areas are delineated from the cells where the maximum water depth exceeds 5 cm.

## 3 Results

### 3.1 Model Runtimes

An overview of the average model runtimes per km^2^ of simulated wet area and the average simulated times for the regions of Austria is given in Table 1.The model runtime is defined as the cumulative wall clock time that a single GPU needs to process the specified area. The simulated time specifies the duration of the simulated timespan, which is given by the duration of the streamflow hydrographs inside the domain. It is larger than average in regions with lakes, for example, in Carinthia and Upper Austria. For a 100‐year flood, the accelerated computational model ensures that a tile is processed with a runtime of a quarter of the maximum hydrograph duration on average. With a cluster of 10 GPUs the actual model runtime is approximately one tenth of the runtime specified in Table 1 resulting in an overall runtime of less than a month for all of Austria.

**Table 1:** Wet Areas, Average Model Runtimes, and Average Simulated Time by Austria's Regions for a 100‐Year Flood

Region	Sim. wet area (km^2^)	Runtime (hours/km^2^)	Simulated time (days)
Carinthia	372.1	2.40	7.80
Lower Austria	1463.7	1.80	7.24
Salzburg	209.4	1.92	5.93
Styria	636.9	1.28	4.71
Tyrol and Vorarlberg	307.5	1.79	4.44
Upper Austria	541.9	2.51	9.87
Austria	3531.5	1.88	6.54

*Note.* For each region, the model runtimes are given in the average values per km^2^ of simulated wet area (per 250,000 wet cells).

**Figure 11:**
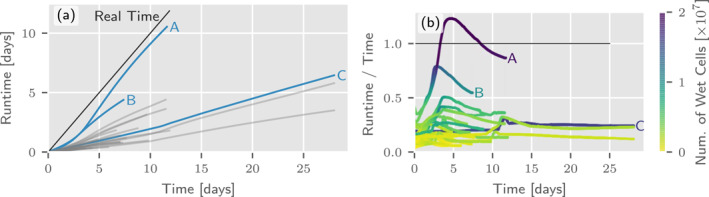
(a) Model runtimes for each of the 25 tiles of Upper Austria for a 100‐year flood. Simulation of a tile is performed on a single graphics processing unit (GPU). (b) Depending on the number of cells and the wave speeds inside these tiles, the simulation of a single tile is between real time and 20 times faster than realtime. The number of wet cells in a tile ranges between 1 and 20 million.

For the tiles of Upper Austria, which are shown in Figure 6, the model runtimes per tile (for one GPU) are displayed in Figure 11a. Tiles that are covered by large inundated areas require longer runtimes, as can be seen when comparing tiles A, B and C in Figure 11b. In tile A, more than 20 million cells are wet, that is, an area of 80 km^2^ is flooded. Only tiles with large lakes differ from the general pattern, for example, in tile C the lake Attersee is responsible for a wet area of 46 km^2^. The ratio between model runtime and simulated time in Figure 11b does not not show any kinks, indicating that no high speeds occur which shows the robustness of the underlying scheme. As a side note, doubling the resolution increases the runtime by a factor of 5–7 times (Horváth et al., 2020). The factor is larger than 4 because of the shorter timestep according to the Courant–Friedrichs–Lewy (CFL) condition.

### 3.2 Comparison With Measured Rating Curves

**Figure 12:**
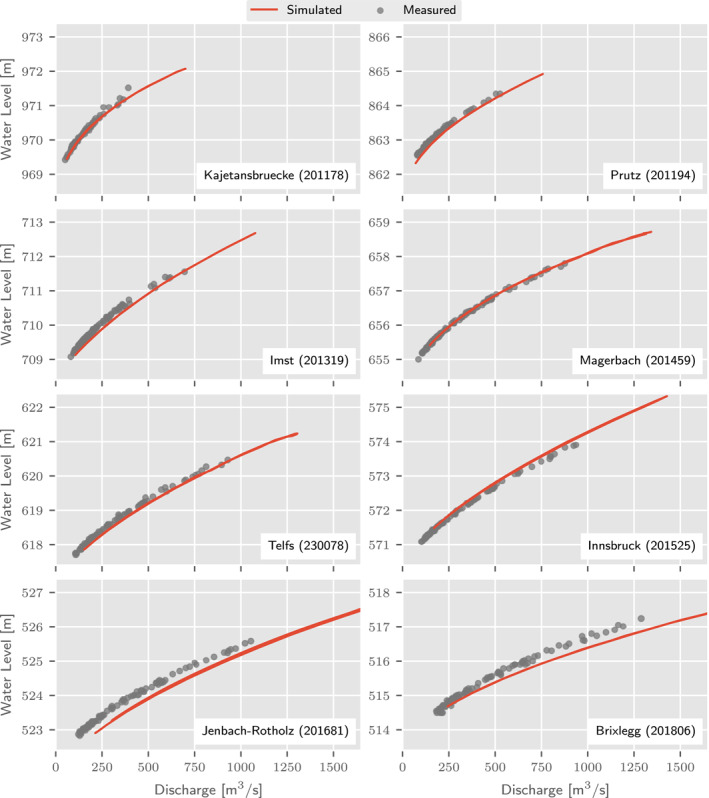
Simulated (red lines) and observed (gray points) discharges and water levels at eight Inn gauges in Tyrol. The reference measurements are monthly maxima collected between 2010 and 2017. Catchment areas of the gauges range from 2,162 to 8,504 km^2^. Station names and numbers are given in the panels.

To validate the accuracy of the simulations, we compare them with measured rating curves at eight stream gauges along the Inn river (Figure 12), where measured river bed profiles exist. Very good agreement is found for the top six gauges and slightly poorer agreement is found for Jenbach‐Rotholz and Brixlegg. The rating curve of Jenbach‐Rotholz shows a bias of around 30 cm which may be due to an inaccurate representation of the measured river profiles in the neighborhood of the gauge. Additional surveys would be needed to shed light on this issue, but this is beyond the scope of this article. At Brixlegg, simulated water levels tend to deviate from measured water levels for high discharges. In this case, an inaccurate representation of the flow at several bridges downstream of the gauge explains the low simulated water levels at high flows, since bridges are not explicitly considered in the simulations. Another reason may be an underestimation of the channel roughness which might be addressed by local recalibration.

### 3.3 Comparison With Local Flood Hazard Maps

For a spatially distributed validation, we compare the simulated inundated areas against local flood hazard zones, which have been developed in local studies for individual river reaches and approved by the respective authorities. Both the local hazard polygons used as reference (*R*) and the simulated flood zones are sampled on a 8 by 8 m^2^ grid. This results in two possible states for each cell in the reference benchmark data, either wet (*R*_1_) or dry (*R*_0_), if the reference polygons covers more or less than half of the respective grid cell. In the model *M*, a cell is considered wet (*M*_1_) if a threshold of 5 cm is exceeded at the cell center, otherwise it is dry (*M*_0_). Four performance measures are used. The first is the hit rate (HTR), defined by the number of cells that are wet in both the model and the reference over the number of wet cells in the reference data, that is,

(6) $$HTR=\frac{\left|M_{1}R_{1}\right|}{\left|R_{1}\right|}\in \left[0,1\right].$$

The hit rate provides information whether the model can correctly replicate the wet cells, ignoring however if the model overpredicts flood extents. The false alarm ratio (FAR) accounts for the overprediction of the flood extent as it is directly proportional to the number of false alarms, that is, cells that are wet in the model but dry in the reference data.

(7) $$FAR=\frac{\left|M_{1}R_{0}\right|}{\left|M_{1}\right|}\in \left[0,1\right].$$

A FAR equal to zero indicates that there are no false alarms. The critical success index (CSI) provides a measure of fit by relating the correctly predicted wet cells to the total number of wet cells in either model or reference or both, that is,

(8) $$CSI=\frac{\left|M_{1}R_{1}\right|}{\left|M_{1}R_{1}+M_{0}R_{1}+M_{1}R_{0}\right|}\in \left[0,1\right].$$

A CSI of one describes a perfect match of the model flood extents and the reference data. Finally, the error bias (EBS) shows if a model tends toward overestimating (EBS greater than one) or underestimating (EBS smaller than one) flood extents. The error bias describes the number of false alarms over the number of cells that the model missed, that is,

(9) $$EBS=\frac{\left|M_{1}R_{0}\right|}{\left|M_{0}R_{1}\right|}\in \left[0,\infty \right].$$

Not all simulated rivers are covered by the reference flood hazard maps, thus a buffer zone around rivers covered in the reference flood polygons is computed. The buffer zone width *w*_*b*_ depends on the catchment area *A*_*c*_, that is, $w_{b}=200+15\sqrt{A_{c}}$. Furthermore, lakes and buildings are excluded in the buffer zone for a fair comparison. Model evaluation was subsequently restricted to this buffer zone.

For Austria, a critical success index (CSI) score of 0.69 and a hit rate of 83% is achieved. In Table 2 these performance measures are evaluated per region. The CSI ranges between 0.61 and 0.74, the highest value is achieved in Styria, the lowest in Salzburg. The hit rate ranges between 0.71 and 0.88, the highest rate is achieved in Lower Austria. The false alarm ratio (FAR) ranges between 0.11 in Styria and 0.24 in Lower Austria. In Lower Austria, hit rate, FAR, and error bias are high due to a modest overestimation of inundated areas in the large floodplains along the Danube and its tributaries. In Styria, the good fit may be explained by the fact that rivers flow mostly through natural floodplains in hilly lowlands or alpine valleys. In Carinthia, Tyrol and Salzburg the floodplains lie in clearly defined alpine valleys where the hinterland is often protected by highways used as levees. These levees frequently have small openings for tributaries or streets, which can be closed if a flood only occurs at the main river, and are therefore mostly considered closed in the reference flood maps. However, in the proposed regional approach used here, they remain open to facilitate free flow of the tributary. Thus, model performance is worse in alpine valleys where rivers are constrained by highways used as levees, for example, Salzburg and Tyrol, than in regions where most levees do not have a secondary purpose, for example, in Lower and Upper Austria. These findings suggest that regional inundation modeling of rivers constrained by complex defensive protection measures is more involved and associated with a higher uncertainty (Annis et al., 2020) than that of rivers flowing through natural floodplains.

**Table 2:** Performance Metrics for Austria's Regions for a 100‐Year Flood

Region	CSI	HTR	FAR	EBS	$A_{R_{1}M_{1}}$ (km^2^)	Max. *A*_*C*_ (km^2^)	Median *A*_*C*_ (km^2^)
Carinthia	0.71	0.84	0.18	1.13	113.8	7065.8	205.2
Lower Austria	0.68	0.88	0.25	2.48	630.7	130804.6	123.7
Salzburg	0.60	0.71	0.21	0.64	54.4	6124.1	181.0
Styria	0.73	0.81	0.12	0.55	380.5	9829.6	95.3
Tyrol and Vorarlberg	0.61	0.76	0.23	0.95	118.0	9531.1	96.2
Upper Austria	0.72	0.81	0.14	0.66	279.3	92515.6	74.7

*Note.* The metrics include the critical success index (CSI), hit rate (HTR), false alarm ratio (FAR), error bias (EBS), the area $A_{M_{1}R_{1}}$ that is wet in both the model and the reference, and the maximum and median of the catchment areas *A*_*C*_.

For a historical reach‐scale scenario with manual calibration of roughness parameters, a CSI value of 0.89 against observed high water marks was reported in Echeverribar et al. (2019). Aronica et al. (2002) report CSI values of 0.7–0.85 for the best pick of ensemble scenarios. Thus, CSI values of 0.9 seem to represent an upper limit for local models. For two continental‐scale models (Alfieri et al., 2014; Wing et al., 2017), CSI values are in the range of 0.44–0.65, and 0.51–0.9, respectively. However, comparisons across regions need to be considered in light of the complexity of the floodplain topography, for example, if the floodplain is defended or developed, as it has an effect on the model performance (Wing et al., 2017).

**Figure 13:**
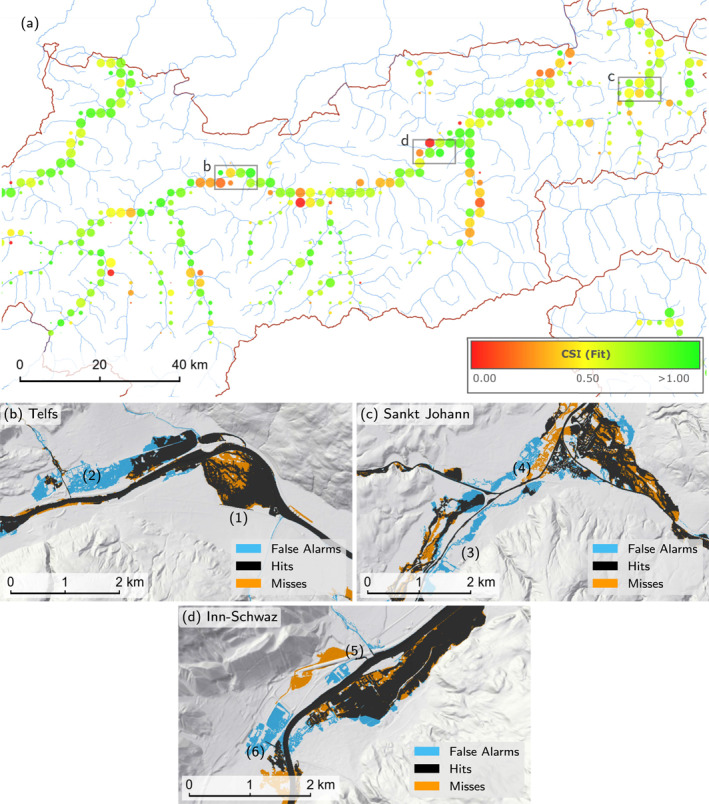
(a) Critical success index (CSI) scores in Tyrol aggregated on a 2.5 by 2.5 km^2^ grid. (b) Both models resolve levees (1) at the Inn around Telfs, but the adopted regional model causes larger inundation areas for the northern tributaries (2). (c) In Sankt Johann, flooded areas deviate due to the proposed regional modeling approach including the adjustments. Upstream of the confluence the flood quantiles are higher than those of the local reference data set resulting in larger modeled indundation areas (3), (4). (d) At the Inn in Schwaz an underpass is included in the reference data set, but not in the DTM used here (5) and vice‐versa (6).

Figure 13a shows the CSI values aggregated on a grid with 2.5 × 2.5 km^2^ cells for Tyrol. Illustrative examples of the inundated areas of the model and the reference data set are shown in Figures 13b–13d. In the following, we give reasons for the differences of the two data sets in order of their importance.

First, the inundated areas of the reference flood hazard maps are constructed for single reaches using simulations that are mass preserving. Typically, in these simulations the discharge of the main stream corresponds to that of the return period of the scenario, while the discharges of the tributary are reduced to the extent to make the confluences mass conserving. Normally, discharges of the tributaries correspond to return periods of 3–5 years, while the main stream maintains a 100‐year flood quantile. The reference areas thus describe a realization of a single possible event. In contrast, the proposed model represents a regional pattern of the ensemble of multiple hypothetical events for a water level consistent with a 100‐year flood quantile everywhere (Figure 8a). Thus, in our simulations inundated areas tend to be larger upstream of confluences, and smaller downstream, than in the reference map. This is particularly apparent in Figures 13b and 13c.

Second, rivers exist that are included in the data set used here but are not included in the reference data set and vice versa. Modeled inundated areas of tributaries are sometimes inside the buffer zone even if they are not included in the reference flood hazard maps. In this case, flooding of small rivers in the present model are incorrectly treated as false alarms.

Third, the DTMs are different as the data source and date of the ALS campaigns are not the same. The reference flood hazard maps include underpasses which are considered closed in the reference but open in our model and vice versa, thus causing differences in the flooded areas as is shown in Figure 13d. In general, levees are resolved and protected areas remain dry (Figure 13b). In rare cases, mobile walls or concrete walls with a width smaller than 2 m, which are resolved in the reference data set but not here, cause an overestimation of wet areas. In general, uncertainties are larger in urban areas, which is also in line with the findings of Dottori et al. (2013); Wing et al. (2017); Annis et al. (2020).

As the reference flood hazard maps are also created from a shallow water model in addition to comparisons with observed flood cases, and some of the data are shared between the models (e. g., river bed), this test is not fully independent and may not reveal all the biases. A validation against remotely observed inundation patterns, observed flood marks or against validation claims of individual inundated buildings could help to further improve the modeling approach (Wing et al., 2021; Zischg et al., 2018).

### 3.4 Model Sensitivity to Tiling

**Figure 14:**
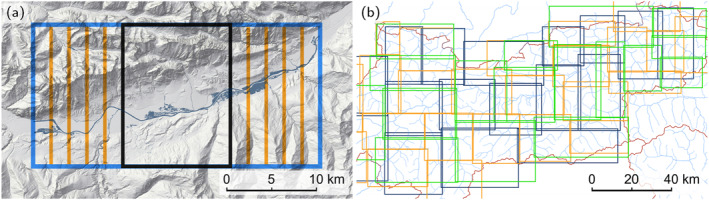
(a) The sensitivity of the inundated areas in the center tile (black) is investigated for the inundated areas of the reference tile (blue) and eight alternative tiles with shrunken eastern or western boundaries (orange). (b) Three different tilings for Tyrol in order to compare the sensitivity of the inundated areas with respect to the domain decomposition.

In this section, we investigate the effect of varying the boundary for a specific tile and the effect of different tilings for an entire region comprising up to 20 tiles. In order to test the sensitivity of the BCs for a single tile, we performed a reference simulation for a 100‐year flood on a large tile with a size of 32 × 15.8 km^2^ and simulations on eight smaller alternative tiles (Figure 14a). These tiles are obtained by shrinking the reference tile at the eastern or western border with a step size of 2 km. Table 3 shows performance metrics of these simulations in comparison to the reference simulation. They are evaluated in the tile in the center with a size of 12 × 15.8 km^2^. The metrics show that there is almost perfect agreement if the boundaries are 2 km away from the reference simulation. If the boundaries move further away from the reference boundaries, the agreement diminishes only slightly to a CSI value of 0.98 for the outflow BC. For the inflow BC, the CSI value drops to 0.85 for one tile. In this case, the inflow BC lies in an urban area and crosses multiple buildings. Once the river floods the urban floodplain, a significant proportion of the water volume is held back resulting in lower flood discharges further downstream and comparably lower CSI and HTR values. In fact, the variation of the inflow BC has a greater effect on the inundated areas than the variation of the outflow BC. This demonstrates that occasionally issues for specific borders might occur in a fully automated tiling process. In general, the very high CSI values indicate an overall robust approach.

**Table 3:** Critical Success Index (CSI), Hit Rate (HTR), and False Alarm Ratio (FAR) for Inundated Areas for Variations of the Western or Eastern Tile Border Shown in Figure 14a, Affecting Either the Main River's Inflow or Outflow Boundary Conditions (BC)

CSI	HTR	FAR	Tile border	Change	Affected BC
0.999	0.999	0.000	West	2 km	Inflow
0.965	1.000	0.035	West	4 km	Inflow
0.851	0.851	0.000	West	6 km	Inflow
0.971	1.000	0.029	West	8 km	Inflow
0.999	1.000	0.000	East	2 km	Outflow
0.999	1.000	0.000	East	4 km	Outflow
0.999	1.000	0.001	East	6 km	Outflow
0.983	0.999	0.016	East	8 km	Outflow

In order to test the sensitivity of different tilings for an entire region (around 10,000 km^2^), we examined three different tilings for a 100‐year flood for Tyrol (Figure 14b). The inundated areas are relatively consistent across the three different tilings with CSI values of 0.89, 0.90 and 0.87. The HTR amounts to 0.96, 0.93 and 0.91. Differences are mostly located close to inflow BCs and downstream of lakes. This indicates that discharges obtained by a hydraulic simulation do not always agree well with the specified flood hydrographs derived from the hydrological data at lake outlets. We also compared the alternative tilings with local flood areas in this region as in Section 3.3 and found CSI values of 0.65 for the base tiling, as well as 0.65 and 0.62 for the alternative tilings. Although different tilings do not result in exactly the same inundated areas, the differences are small.

## 4 Discussion

Previous large‐scale flood hazard simulations have often used coupled 1D/2D models (Falter et al., 2016) or resolutions too coarse to explicitly resolve fine structures, for example, levees and small rivers, in the terrain model (Alfieri et al., 2014; Wing et al., 2017). A resolution of 2 m as adopted in this study allows for a direct incorporation of such terrain features. As a consequence, additional techniques, such as subgrid simulation or downsampling algorithms (Neal et al., 2012; Schumann et al., 2014), are not required. Due to the high resolution, openings in levees, for example, street underpasses, can be represented explicitly. This opens up new questions regarding the modeling of underpasses since, without additional information, it is not clear from the DTM whether they will be closed or not in an emergency case. Overall, the direct inclusion of levees and buildings enables a detailed representation of flow in urban regions. The high detail also allows for the direct estimation of building damage and other socio‐economic impacts (Ernst et al., 2010).

In these types of large‐scale applications, the storage capacities needed for the entire domain almost always exceeds what can be accomodated on a single computational device, so some tiling is necessary. One option is to couple the subdomains through overlapping halo and ghost cells and perform tightly coupled simulations where the state variables are exchanged at every simulation time step (Morales‐Hernández et al., 2021; Xia et al., 2019). For example, Xia et al. (2019) distributed one simulation run for a catchment with a size of 2,500 km^2^ and a resolution of 5 m (i. e., 100 million cells) on several GPUs. The tightly coupled approach tends to increase runtimes as the global timestep is restricted by the highest numerical speed in all the subdomains due to the CFL condition. This introduces a synchronisation barrier at every time step of the hydraulic simulation. Moreover, when simulating multiple non‐overlapping catchments, every catchment needs to be simulated for the maximum flood duration of the entire region. Thus, the runtime tends to increase even further with the number of subdomains. While these issues are avoided in the proposed less tightly coupled approach, one drawback are differences in regions where the tiles overlap. As the domain is subdivided into tiles, each of which is simulated independently and in parallel on one GPU, state variables are not synchronized during the simulation across the tiles. However, the inundated areas in different tiles are aligned with each other through consistent BCs, which are defined in a unique way for any river position and are thus independent of the tiling. The analysis in Section 3.4 shows that, although the inundated areas depend on the tiling, the resulting differences are small. We therefore consider the proposed less tightly coupled approach an interesting alternative to tightly coupled approaches.

In the proposed model the region of interest is tesselated into rectangular tiles rather than into individual river reaches, as for example, in Bates et al. (2021); Sampson et al. (2015); Wing et al. (2017). The proposed approach allows the simulation of flood discharges associated with a consistent return period across all river reaches. The inundated areas are determined by the flood quantiles representing the flood peak for a given return period at every node in the stream network. As a consequence, the simulated flood areas do not correspond to a single observable event, but are related to observed, real events through the flood frequency curve and associated flood discharges along the stream network. We consider this an attractive alternative to long‐term stochastic simulations (e. g., Falter et al., 2015), because of the much faster runtimes. The application of a probabilistic sampling approach (e. g., with copulas (Bender et al. (2016)) to numerous confluences in ungauged catchments requires thousands of simulations which would render the spatial resolution adopted here unfeasible.

Although the modeling framework is mostly automatic, we integrate manual methods where an improvement over the automatic methods was deemed feasible. The manual steps are pre‐processing steps before the simulation. They involve correction of data errors in the terrain model, the river network, for example, manual correction of the river and river bank lines (Wimmer et al., 2021), and the river bed geometry, for example, obstacle removal. The flood discharges are checked manually and, if necessary, corrected to account for local hydrological particularities (R. Merz & Blöschl, 2005). Lastly, the simulation tiles are specified manually to balance the factors mentioned in Section 2.3.1.

## 5 Conclusions

In this paper, we present a modeling framework for large‐scale hydraulic simulations of river floods. With a focus on accuracy and simulation speed, we employ a second‐order finite‐volume scheme that discretizes the full, transient shallow water equations (Buttinger‐Kreuzhuber et al., 2019) implemented on graphics processing units (GPUs). Inundated areas are simulated for the whole of Austria (84,000 km^2^) with quadratic cells of 2 × 2 m^2^. We achieve local relevance through the number of included streams and through the high resolution of the DTM and the simulation grid, which allows explicit representation of dams, buildings and small rivers. Inflow boundary conditions (BCs) are automatically prescribed from hydrologic data. Hydraulic submodels of high‐head power plants and culverts provide an accurate description of local effects. Dynamic outflow BCs account for instationary two‐dimensional effects, for example, from retention basins.

In order to efficiently map the inundated areas, we propose a workload distribution based on simulation tiles that allows adaptation to the capabilities of the individual computational devices. The approach allows for arbitrarily placed tiles with a domain size not constrained by hydrological data, but only bound by current hardware limitations. For tiles with sizes of around 600 km^2^, the GPU‐accelerated robust hydraulic engine ensures fast hydrodynamic simulations. For all of Austria, 182 tiles are processed in parallel on a distributed setup of 10 GPUs. The effective runtime for the entire region of Austria is less than a month for a 100‐year flood simulation, which results in 3,532 km^2^ of inundated areas or 883 million wet pixels.

By providing additional adjustment source terms to the hydraulic engine, a novel approach to maintain consistent flood return periods across the river network is presented. With these adjustments, we are able to simulate all rivers in a tile in one run. The adjustments violate mass conservation at confluences, thus results deviate from conventional mass‐conserving flood hazard maps. This approach provides an efficient way to map inundated areas at confluences without the need for ensemble simulations.

The automation framework Visdom (Waser et al., 2011) controls the complete dataflow including the automated generation of the inputs to the hydraulic simulations, their execution, and the post‐processing without the need for manual interventions. Manual work is only required for a few specific tasks, for example, tiling of the region of interest and providing data corrections. A flexible setup ensures that changes and manual corrections are automatically integrated and propagated forward to the simulations.

We regard the presented model as a prototype for a new standard that brings local relevance to large‐scale high resolution modeling. The simulated rating curves show good agreement at stream gauges when compared with measured rating curves. The model delivers flood hazard maps comparable to flood hazard maps created in local studies with a critical success index (CSI) score of 0.69 and a hit rate of 83% across Austria. The individual CSI scores across Austria's regions range from 0.61 to 0.74. Deviations from local reference maps occur due to the consistent flood return periods or due to differences in the DTM, for example, open underpasses. In future work, uncertainties in the model cascade and the effects of climate change could be addressed. One possibility would be ensembles of simulations that investigate changing streamflow conditions from climate projections.

## Conflict of Interest

The authors declare no conflicts of interest relevant to this study.

## Data Availability

The flood hazard maps of the presented model are available at https://www.hora.gv.at from Autumn 2021. The local flood hazard maps used as reference data set in Tyrol are available at https://data-tiris.opendata.arcgis.com/datasets/ueberflutungsflaechen-1. The version from 29 January 2021 was used. Additional data used during the study were provided by a third party. Direct requests for the terrain data and the river network may be made to the provider, the BMLRT (https://www.bmlrt.gv.at).
